# SARS-CoV-2 antibody-positivity protects against reinfection for at least seven months with 95% efficacy

**DOI:** 10.1016/j.eclinm.2021.100861

**Published:** 2021-04-28

**Authors:** Laith J. Abu-Raddad, Hiam Chemaitelly, Peter Coyle, Joel A. Malek, Ayeda A. Ahmed, Yasmin A. Mohamoud, Shameem Younuskunju, Houssein H. Ayoub, Zaina Al Kanaani, Einas Al Kuwari, Adeel A. Butt, Andrew Jeremijenko, Anvar Hassan Kaleeckal, Ali Nizar Latif, Riyazuddin Mohammad Shaik, Hanan F. Abdul Rahim, Gheyath K. Nasrallah, Hadi M. Yassine, Mohamed Ghaith Al Kuwari, Hamad Eid Al Romaihi, Mohamed H. Al-Thani, Abdullatif Al Khal, Roberto Bertollini

**Affiliations:** a Infectious Disease Epidemiology Group, Weill Cornell Medicine-Qatar, Cornell University, Doha, Qatar; b World Health Organization Collaborating Centre for Disease Epidemiology Analytics on HIV/AIDS, Sexually Transmitted Infections, and Viral Hepatitis, Weill Cornell Medicine–Qatar, Cornell University, Qatar Foundation, Education City, Doha, Qatar; c Department of Population Health Sciences, Weill Cornell Medicine, Cornell University, New York, NY, United States; d Hamad Medical Corporation, Doha, Qatar; e Genomics Laboratory, Weill Cornell Medicine-Qatar, Cornell University, Doha, Qatar; f Department of Genetic Medicine, Weill Cornell Medicine-Qatar, Cornell University, Doha, Qatar; g Department of Mathematics, Statistics, and Physics, Qatar University, Doha, Qatar; h College of Health Sciences, QU Health, Qatar University, Doha, Qatar; i Biomedical Research Center, Member of QU Health, Qatar University, Doha, Qatar; j Department of Biomedical Science, College of Health Sciences, Member of QU Health, Qatar University, Doha, Qatar; k Primary Health Care Corporation, Doha, Qatar; l Ministry of Public Health, Doha, Qatar

**Keywords:** SARS-CoV-2, Epidemiology, Reinfection, Immunity, Genetics

## Abstract

**Background:**

Reinfection with the severe acute respiratory syndrome coronavirus 2 (SARS-CoV-2) has been documented, raising public health concerns. SARS-CoV-2 reinfections were assessed in a cohort of antibody-positive persons in Qatar.

**Methods:**

All SARS-CoV-2 antibody-positive persons from April 16 to December 31, 2020 with a PCR-positive swab ≥14 days after the first-positive antibody test were investigated for evidence of reinfection. Viral genome sequencing was conducted for paired viral specimens to confirm reinfection. Incidence of reinfection was compared to incidence of infection in the complement cohort of those who were antibody-negative.

**Findings:**

Among 43,044 antibody-positive persons who were followed for a median of 16.3 weeks (range: 0–34.6), 314 individuals (0.7%) had at least one PCR positive swab ≥14 days after the first-positive antibody test. Of these individuals, 129 (41.1%) had supporting epidemiological evidence for reinfection. Reinfection was next investigated using viral genome sequencing. Applying the viral-genome-sequencing confirmation rate, the incidence rate of reinfection was estimated at 0.66 per 10,000 person-weeks (95% CI: 0.56–0.78). Incidence rate of reinfection versus month of follow-up did not show any evidence of waning of immunity for over seven months of follow-up. Meanwhile, in the complement cohort of 149,923 antibody-negative persons followed for a median of 17.0 weeks (range: 0–45.6), incidence rate of infection was estimated at 13.69 per 10,000 person-weeks (95% CI: 13.22–14.14). Efficacy of natural infection against reinfection was estimated at 95.2% (95% CI: 94.1–96.0%). Reinfections were less severe than primary infections. Only one reinfection was severe, two were moderate, and none were critical or fatal. Most reinfections (66.7%) were diagnosed incidentally through random or routine testing, or through contact tracing.

**Interpretation:**

Reinfection is rare in the young and international population of Qatar. Natural infection appears to elicit strong protection against reinfection with an efficacy ~95% for at least seven months.

**Funding:**

Biomedical Research Program, the Biostatistics, Epidemiology, and Biomathematics Research Core, and the Genomics Core, all at Weill Cornell Medicine-Qatar, the Ministry of Public Health, Hamad Medical Corporation, and the Qatar Genome Programme.

**Research in context**

**Evidence before this study**

The severe acute respiratory syndrome coronavirus 2 (SARS-CoV-2) infection has spread worldwide, causing disease and mortality, as well as social disruption and economic loss. In addition to the risk of first infection, reinfection during this prolonged pandemic has raised additional public health concerns. PubMed and medRxiv preprint searches were updated on March 24, 2021 using the search criteria ("SARS-CoV-2″ OR "COVID-19″) AND “reinfection.” These searches identified eight cohort studies providing estimates for the efficacy of natural infection against reinfection. These studies were conducted in Austria, Denmark, Qatar, Switzerland, the UK, and the USA, and reported an efficacy against reinfection ranging between 80 and 95%.

**Added value of this study**

The study assessed cumulative risk of SARS-CoV-2 reinfection and incidence rate of reinfection in a nationwide cohort of 43,044 antibody-positive individuals who were followed for up to 35 weeks. The total cohort follow-up time exceeded 600,000 person-weeks, which is comparable to or greater than the follow-up time of COVID-19 vaccine trials. The study demonstrated and confirmed through viral genome sequencing that SARS-CoV-2 reinfection does occur, but only rarely, with the cumulative risk of reinfection being ~2 per 1000 persons after 35 weeks of follow-up, and the incidence rate of reinfection estimated at <1 per 10,000 person-weeks. Meanwhile, in the complement cohort of 149,923 antibody-negative persons, the cumulative risk of infection was much higher, estimated at ~31 per 1000 persons after 46 weeks of follow-up, and the incidence rate of infection was estimated at ~14 per 10,000 person-weeks. Efficacy of natural infection against reinfection was estimated at 95%. The study showed that there was no evidence for waning of protective immunity against reinfection in this cohort for over 7 months.

**Implications of all the available evidence**

There is concrete evidence that reinfection can occur in individuals with detectable antibodies for SARS-CoV-2 infection, even in some with high antibody titers. However, the occurrence of reinfection is rare for over 7 months of follow-up after the first antibody-positive test and with no sign of waning of protective immunity. These findings suggest that induced SARS-CoV-2 immunity, whether induced through natural infection or vaccination, is very efficacious against infection (>90%) and may persist for at least 7 months. The findings also suggest that prioritizing vaccination for those who are antibody-negative, as long as doses of the vaccine remain in short supply, could enhance the health, societal, and economic gains achieved by vaccination.

Alt-text: Unlabelled box

## 1 Introduction

The severe acute respiratory syndrome coronavirus 2 (SARS-CoV-2) pandemic has caused extensive disease and death, with social and economic losses [1], [2], [3], [4]. In addition to the risk of first infection, reinfection during this prolonged pandemic has raised additional public health concerns [5], [6], [7], [8], [9].

We previously assessed the cumulative risk and incidence rate of documented reinfection in a cohort of 130,266 SARS-CoV-2 polymerase chain reaction (PCR)-confirmed infected persons in Qatar [5], a country of 2.8 million people [10,11] that experienced a large SARS-CoV-2 epidemic [12], [13], [14], [15], [16]. Benefiting from a centralized data-capture system for nationwide SARS-CoV-2 PCR testing and using viral genome sequencing, we quantified the cumulative risk of reinfection at ~2 reinfections per 10,000 infected persons [5]. Incidence rate of reinfection was estimated at 0.36 (95% CI: 0.28–0.47) per 10,000 person-weeks [5].

Serological testing for SARS-CoV-2 infection has been expanding in Qatar [14,16,17]. The first objective of the present study was to quantify the cumulative risk and incidence rate of documented reinfection in a cohort of 43,044 persons who had a *laboratory-confirmed, anti-SARS-CoV-2 positive result*, regardless of whether these persons had ever had a diagnosed PCR-confirmed infection. Persons with a *PCR-confirmed* infection could, in principle, be *biologically* different from persons with an *antibody-confirmed* infection, as the former population is more likely to have experienced a symptomatic or even serious primary infection, while the latter population is more likely to have experienced an asymptomatic or mild primary infection that may never have been diagnosed. Moreover, some of those with PCR-confirmed infection may not have developed detectable antibodies [5,7]. In an earlier study in Qatar, we found that 9% of those who were PCR positive >3 weeks before the serology test were antibody negative [12]. The second objective was to estimate the efficacy of natural infection against reinfection by comparing the incidence rate of reinfection to the incidence rate of infection in the complement cohort of 149,923 persons who had a *laboratory-confirmed, anti-SARS-CoV-2 negative result*.

The present study thus provides an independent assessment of the risk of reinfection in a *biologically* different population from that of PCR-confirmed infected persons. A major strength of the present study is the long follow-up time of each antibody-positive person in this cohort, which had a median of 16.3 weeks for a total cohort follow-up time of 610,832.6 person-weeks, comparable to or greater than the follow-up time in COVID-19 vaccine trials [18], [19], [20]. An added strength is the comparison to the incidence rate of infection in a large cohort of antibody-negative persons with a similar follow-up time. The study therefore allows assessment of reinfection for more than seven months after primary infection, and provides empirical evidence for possible effects of any waning of immunity.

## 2 Methods

### 2.1 Sources of data

We analyzed the centralized, integrated, and standardized national anti-SARS-CoV-2 serological testing database compiled at Hamad Medical Corporation (HMC), the main public healthcare provider and the nationally designated provider for Coronavirus Disease 2019 (COVID-19) healthcare needs. The database covers essentially all serological testing for SARS-CoV-2 conducted in Qatar, including both testing done on residual blood specimens collected for routine clinical care from attendees at HMC [17] and during a series of population-based serological surveys [14,16]. Most serological testing was done on the residual clinical care specimens and tested individuals were not aware of the testing result, nor was the serological result used for case management. The tested population is broadly representative of the urban population of Qatar [17], but less so of the craft and manual workers population who typically receive their primary healthcare at Qatar Red Crescent Society centers [14]. Qatar launched its vaccination campaign on December 21, 2020 [21], around the time this study was concluded (December 31, 2020), so very few individuals had been vaccinated at time of this study.

The antibody database was linked to the HMC national SARS-CoV-2 PCR testing and COVID-19 hospitalization and severity database [22]. The latter includes records for all SARS-CoV-2 PCR testing conducted in Qatar since the start of the epidemic. The database also includes all COVID-19 hospitalizations and their infection severity classifications, assessed through individual chart reviews by trained medical personnel following World Health Organization (WHO) guidelines [23]. Antibody data were also linked to the centralized COVID-19 death registry, which includes all COVID-19 deaths assessed per WHO guidelines [24]. The STROBE statement checklist can be found in Supplementary Table S1.

### 2.2 Laboratory methods

Antibodies against SARS-CoV-2 in serological samples were detected using the Roche Elecsys® Anti-SARS-CoV-2 assay (Roche, Switzerland), an electrochemiluminescence immunoassay that uses a recombinant protein representing the nucleocapsid (N) antigen for antibody binding. Results were interpreted according to the manufacturer's instructions (reactive: optical density (proxy for antibody titer [25]) cutoff index ≥1.0 vs. non-reactive: optical density cutoff index <1.0).

Nasopharyngeal and/or oropharyngeal swabs (Huachenyang Technology, China) were collected for PCR testing and placed in Universal Transport Medium (UTM). Aliquots of UTM were: extracted on the QIAsymphony platform (QIAGEN, USA) and tested with real-time reverse-transcription PCR (RT-qPCR) using TaqPath™ COVID-19 Combo Kits (Thermo Fisher Scientific, USA) on an ABI 7500 FAST (Thermo Fisher, USA). Samples were extracted using a custom protocol [26] on a Hamilton Microlab STAR (Hamilton, USA) and tested using AccuPower SARS-CoV-2 Real-Time RT-PCR Kits (Bioneer, Korea) on an ABI 7500 FAST, or loaded directly into a Roche cobas® 6800 system and assayed with a cobas® SARS-CoV-2 Test (Roche, Switzerland). The first assay targets the viral S, N, and ORF1ab regions. The second targets the virus’ RdRp and E-gene regions, and the third targets the ORF1ab and E-gene regions. All testing was conducted at HMC Central Laboratory or at Sidra Medicine Laboratory, following standardized protocols.

### 2.3 Suspected reinfection case eligibility and classification

Reinfection was defined as a PCR positive result in an individual who had a prior infection that had cleared. All SARS-CoV-2 antibody-positive persons in Qatar with at least one PCR-positive swab that occurred ≥14 days *after* the first-positive antibody test were considered *suspected cases* of reinfection. These were classified as showing either *good* evidence, *some* evidence, or *weak* (or *no*) evidence for reinfection based on criteria applied to each case (Table 1). We defined the *reinfection swab* as the first-positive PCR swab that was identified ≥14 days after the first-positive antibody test. The 14-day cutoff was incorporated to exclude cases in which antibody testing and PCR testing were done around the same time, as part of clinical care of COVID-19 patients. A PCR-positive swab within a few days of an antibody-positive test is likely to reflect active primary infection under clinical consideration rather than a reinfection.

**Table 1:** Classification of suspected cases of SARS-CoV-2 reinfection based on the strength of supporting epidemiological evidence.

Cases of SARS-CoV-2 reinfection	Definition
Suspected cases of SARS-CoV-2 reinfection	All antibody-positive persons with at least one PCR-positive swab that occurred ≥14 days after the first-positive antibody test
*Good* evidence for reinfection	Individuals who had a PCR-positive swab with a Ct value ≤30 at least 14 days after the first-positive antibody test and who had not had a PCR-positive swab within the 45 days preceding the reinfection swab
*Some* evidence for reinfection	Individuals who had a PCR-positive swab with a Ct value >30 at least 14 days after the first-positive antibody test and who had not had a PCR-positive swab within the 45 days preceding the reinfection swab
*Weak* evidence for reinfection	Individuals who had a PCR-positive swab at least 14 days after the first-positive antibody test, but who had one or more PCR-positive swabs within the 45 days preceding the reinfection swab

Ct, cycle threshold; PCR, polymerase chain reaction.

Suspected reinfection cases with a PCR cycle threshold (Ct) value ≤30 for the reinfection swab (suggestive of a recent active infection) [27], [28], [29] and who had *not* had a PCR-positive swab for 45 days preceding the reinfection swab (to rule out persistent PCR positivity due to non-viable virus fragments) [5,27,[30], [31], [32]], were considered as showing *good evidence for reinfection*. The decision to use a 45-day duration, instead of a longer duration, was a conservative choice. Had we set this duration for 60 or 90 days, for example, we would have missed some identified reinfections. Suspected reinfection cases who had not had a PCR-positive swab for 45 days preceding the reinfection swab, but whose Ct value for the reinfection swab was >30, were considered as showing *some evidence for reinfection*. Suspected reinfection cases who *had* a PCR-positive swab within 45 days preceding the reinfection swab were considered as showing *weak* (or *no*) evidence for reinfection, as they were likely to reflect prolonged PCR positivity of the primary infection rather than a reinfection [5,27,[30], [31], [32]].

### 2.4 Viral genome sequencing and analysis

For a subset of investigated reinfection cases with *good* or *some evidence for reinfection*, there were records indicating prior diagnosis of the primary infection. Viral genome sequencing was thus conducted to confirm reinfection in this subset of cases whenever it was possible to retrieve both the first-infection PCR-positive swab and the reinfection swab. Details of viral genome sequencing methods are provided in Supplementary Text S1.

### 2.5 Reinfection risk and rate

The Kaplan–Meier curve was used to estimate the cumulative risk of documented reinfection, that is, the proportion of cases with *good or some evidence for reinfection* among all eligible individuals with an antibody-positive test. *Incidence rate* of documented reinfection was calculated by dividing the number of cases with *good* or *some* evidence for reinfection by the number of person-weeks contributed by all anti-SARS-CoV-2 positive cases. The latter was estimated using a Poisson log-likelihood regression model with the STATA 16.1 [33] *stptime* command. The follow-up person-time was calculated starting 14 days after the first-positive antibody test until the reinfection swab, all-cause death, or end-of-study censoring (on December 31, 2020). The temporal trend in incidence rate was assessed with the Mantel-Haenszel method using the STATA 16.1 [33] *stmh* command. Adjusted estimates for the cumulative risk of reinfection and the incidence rate of reinfection were derived by applying the confirmation rate obtained from the viral genome sequencing analysis. Sensitivity analyses were conducted.

### 2.6 Comparator antibody-negative group and efficacy of natural infection against reinfection

SARS-CoV-2 incidence was also assessed in the complement cohort, including all those who tested SARS-CoV-2 antibody-negative in Qatar, to provide an antibody-negative comparator and to assess the efficacy of natural infection against reinfection.

Both cumulative risk of documented infection and incidence rate of documented infection in this antibody-negative cohort were assessed as described above for the antibody-positive cohort, but with the *event* defined here as the first PCR-positive swab that is ≥14 days after the first *antibody-negative* test.

The *efficacy* of natural infection against reinfection was estimated by comparing the incidence rate of reinfection in the antibody-positive cohort to the incidence rate of infection in the comparator antibody-negative cohort:

$$\text{Efficacy}\;\text{against}\;\text{reinfection}=1-\frac{\text{incidence rate of reinfection among the antibody-positive individuals}}{\text{incidence rate of infection mong the antibody-negative individuals}}.$$

### 2.7 Ethical approval

This study was approved by the HMC and Weill Cornell Medicine-Qatar Institutional Review Boards.

### 2.8 Role of the funding source

The funders of the study had no role in study design, data collection, data analysis, data interpretation, or writing of the article.

## 3 Results

### 3.1 Epidemiological analysis

**Fig. 1:**
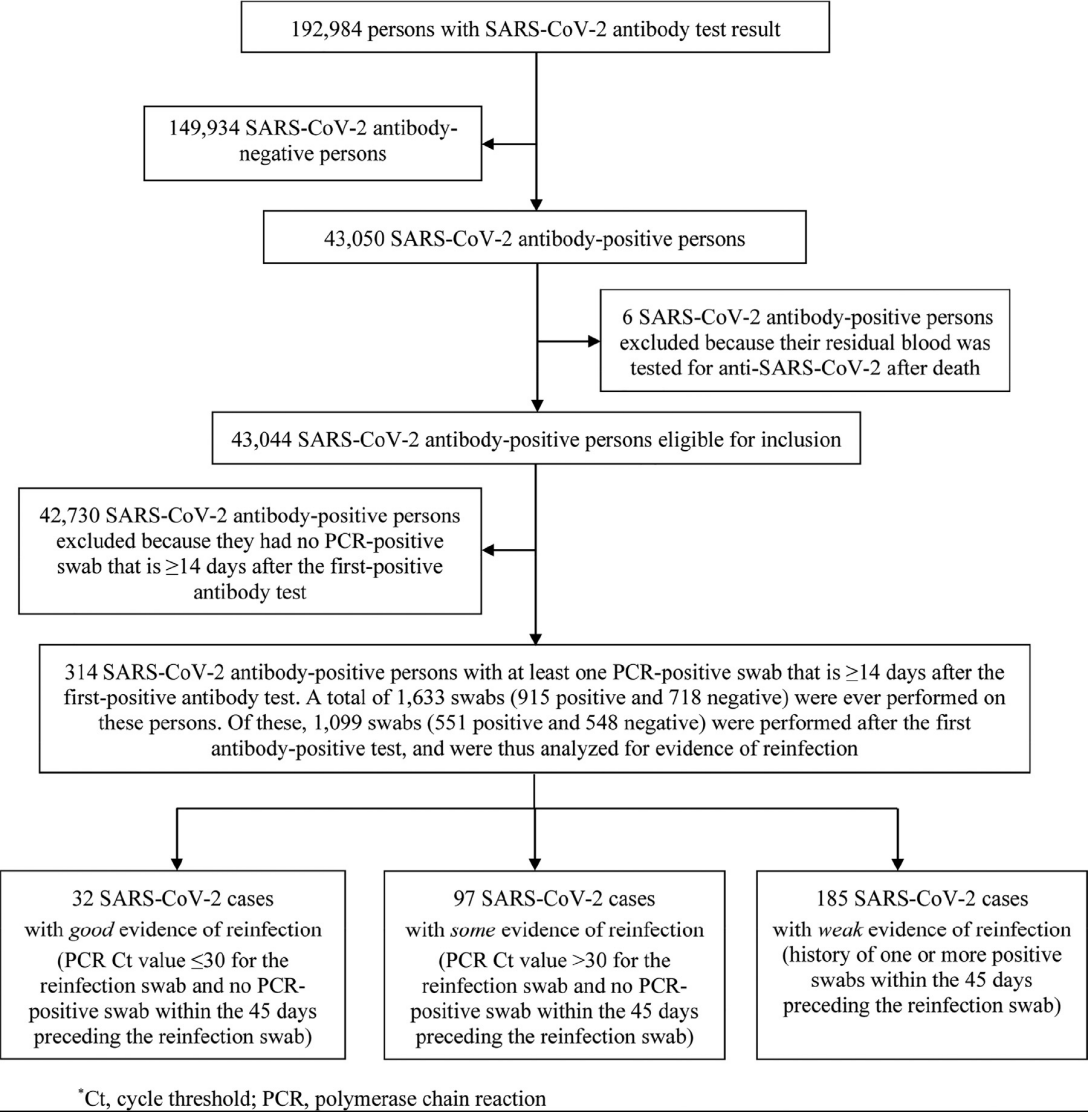
Flowchart describing the selection process of suspected cases of SARS-CoV-2 reinfection and summarizing the results of their reinfection status evaluation.

The process for selecting suspected cases of SARS-CoV-2 reinfection is shown in Fig. 1, which summarizes results of their reinfection status evaluation. Of 192,984 persons tested for anti-SARS-CoV-2 using blood specimens collected between April 16, 2020 and December 31, 2020, 149,934 had negative test results, and were excluded. Six of the remaining 43,050 antibody-positive persons were also excluded because their residual blood was tested for SARS-CoV-2 antibodies after death. This yielded a retrospective cohort of 43,044 antibody-positive persons for whom possible reinfection was assessed.

The cohort included 8953 (20.8%) women and 34,091 men (79.2%) of 158 nationalities. Median age was 35 years for women (interquartile range (IQR): 28–45 years) and 38 years for men (IQR: 31–47 years). Of this cohort, 80.7% had received a PCR test with an overall testing frequency of 1.9 tests per person, and of 0.5 tests per person after the first antibody-positive test. Only 19,976 (46.4%) of these persons had ever had a PCR-positive swab *preceding* their first-positive antibody test. Individual time of follow-up ranged between 0 days and 34.6 weeks, with a median of 16.3 weeks.

Only 314 persons had a PCR-positive swab ≥14 days after the first-positive antibody test, and thus qualified for inclusion in the analysis. There were 1633 swabs (915 positive and 718 negative) collected from these 314 persons, and of these, 1099 (551 positive and 548 negative) were collected *after* the first-positive antibody test.

Investigation of these 314 suspected cases of reinfection yielded 32 cases with *good* evidence for reinfection (Ct ≤30 for reinfection swab), 97 cases with *some* evidence (Ct >30 for reinfection swab), while evidence was *weak* for the remaining 185 cases.

Characteristics of the 129 cases with *good* or *some* evidence for reinfection are shown in Table 2. These individuals had a median age of 37 years (range: <1–72 years) and included 92 men (71.3%). The median time between the *first-positive antibody* test and the *reinfection* swab was 52 days (range: 15–212 days). The median Ct value of the reinfection swab was 32.9 (range: 13.9–38.3). Slightly over a third of cases were diagnosed based on clinical suspicion (*n* = 34; 26.4%) or individual request (*n* = 9; 7.0%), while the rest (*n* = 86) were identified incidentally either through random PCR-testing campaigns/surveys (*n* = 47; 36.4%), through healthcare routine testing (*n* = 18; 14.0%), through contact tracing (*n* = 15; 11.6%), or at a port of entry (*n* = 6; 4.7%).

**Table 2:** Characteristics of individuals classified as showing *good* or *some* evidence of reinfection.

	Demography		Ab testing		PCR testing			
ID#	Sex	Age group	First-positive Ab test date	Ab test optical density (Ab titers)	Reinfection swab date	Average Ct value*	Reason for swab	Presence of symptoms
Good evidence for reinfection								
1	Female	10–14	28 Jul	1.1	29 Sep	21.7	Clinical suspicion§	Yes
2	Female	20–24	02 Jul	1.2	01 Oct	16.6	Contact tracing	Yes
3	Male	50–54	24 Aug	1.2	12 Oct	22.0	Clinical suspicion§	Yes
4	Female	25–29	21 July	1.4	24 Aug	30.0	Individual request†	No
5	Female	40–44	07 Jul	2.0	20 Sep	22.2	Individual request†	Yes
6	Male	30–34	21 Jul	2.1	30 Sep	29.5	Clinical suspicion§	No††
7	Female	30–34	09 Aug	2.3	12 Oct	21.8	Clinical suspicion§	Yes
8	Female	40–44	03 July	2.4	08 Aug	20.5	Port of entry‡	No
9	Female	20–24	09 Aug	2.7	06 Nov	20.5	Contact tracing	No
10	Male	30–34	16 Aug	3.0	22 Sep	28.1	Clinical suspicion§	No††
11	Female	30–34	02 Aug	3.0	27 Dec	28.0	Clinical suspicion§	Yes
12	Male	40–44	13 Jul	4.8	07 Aug	22.6	Survey⁎⁎	No
13	Male	35–39	21 Jun	5.6	14 Sep	23.3	Survey⁎⁎	Not indicated
14	Male	30–34	16 Jul	7.6	17 Sep	29.5	Clinical suspicion§	No††
15	Female	40–44	03 Jul	7.7	16 Sep	23.4	Contact tracing	No
16	Female	30–34	23 Aug	8.7	23 Dec	13.9	Survey⁎⁎	Not indicated
17	Male	55–59	28 Jun	8.8	12 Aug	26.4	Clinical suspicion§	No††
18	Male	50–54	13 Jul	9.2	16 Nov	29.8	Individual request†	No
19	Male	40–44	04 Jul	11.3	12 Oct	28.1	Clinical suspicion§	Yes
20	Male	35–39	09 Jul	11.3	15 Sep	28.1	Contact tracing	Not indicated
21	Male	35–39	03 Nov	14.9	26 Dec	28.8	Survey⁎⁎	No
22	Female	0–9	05 Jul	16.7	17 Sep	29.5	Clinical suspicion§	Yes
23	Male	40–44	20 Aug	22.2	07 Dec	24.4	Port of entry‡	No
24	Female	25–29	27 Aug	24.2	06 Oct	29.5	Clinical suspicion§	No††
25	Female	20–24	25 Aug	25.9	30 Sep	29.1	Survey⁎⁎	No
26	Male	65–69	01 Jun	28.3	22 Jun	27.7	Clinical suspicion§	Not indicated
27	Male	50–54	26 Jun	32.0	23 Sep	29.2	Healthcare routine testing	No
28	Male	65–69	30 Oct	55.9	27 Dec	29.4	Healthcare routine testing	No
29	Male	35–39	13 Jul	75.4	18 Aug	37.6	Survey⁎⁎	No
30	Male	55–59	23 Aug	85.6	12 Dec	27.8	Survey⁎⁎	No
31	Female	30–34	02 Aug	60.1	06 Oct	29.1	Individual request†	Yes
32	Male	20–24	11 Aug	140.0	28 Aug	30.0	Clinical suspicion§	No††

**Some evidence of reinfection**								

33	Female	40–44	23 Jun	1.1	26 Jul	36.2	Survey⁎⁎	No
34	Male	20–24	12 Aug	1.1	11 Sep	NR	Contact tracing	No
35	Male	30–34	16 Jul	1.2	18 Nov	NR	Clinical suspicion§	Yes
36	Male	25–29	21 Oct	1.6	17 Nov	NR	Survey⁎⁎	No
37	Male	30–34	07 Jul	1.7	01 Sep	NR	Clinical suspicion§	No††
38	Female	45–49	05 Jul	2.0	28 Aug	NR	Healthcare routine testing	No
39	Female	65–69	06 Jul	2.0	24 Aug	NR	Survey⁎⁎	No
40	Male	60–64	12 Jul	2.5	08 Oct	NR	Healthcare routine testing	No
41	Female	40–44	20 Jun	3.4	24 Aug	NR	Survey⁎⁎	Not indicated
42	Male	35–39	18 Aug	3.7	08 Nov	NR	Clinical suspicion§	No††
43	Male	45–49	19 Jul	3.9	24 Aug	30.5	Clinical suspicion§	No††
44	Female	20–24	24 Aug	4.1	12 Sep	35.9	Survey⁎⁎	No
45	Female	45–49	22 Oct	4.5	24 Dec	31.0	Clinical suspicion§	Yes
46	Male	60–64	19 Jun	5.2	23 Aug	NR	Clinical suspicion§	No††
47	Male	50–54	28 Jun	5.7	16 Sep	31.3	Port of entry‡	No
48	Female	40–44	26 Aug	6.2	11 Sep	33.9	Port of entry‡	Yes
49	Male	35–39	09 Jun	6.3	05 Oct	31.5	Survey⁎⁎	No
50	Male	25–29	12 Jul	6.9	08 Oct	32.8	Clinical suspicion§	No††
51	Male	50–54	22 Jul	7.6	19 Aug	36.4	Survey⁎⁎	No
52	Male	50–54	30 Jun	7.7	11 Oct	NR	Contact tracing	No
53	Male	35–39	11 Aug	7.9	14 Dec	NR	Survey⁎⁎	No
54	Male	40–44	24 Jun	8.0	05 Sep	34.1	Survey⁎⁎	No
55	Female	25–29	11 Aug	9.0	26 Aug	34.2	Healthcare routine testing	Not indicated
56	Male	40–44	28 Jun	9.9	10 Aug	32.9	Survey⁎⁎	No
57	Female	30–34	15 Aug	10.8	30 Oct	30.2	Clinical suspicion§	No††
58	Male	25–29	21 Jul	11.0	25 Aug	37.4	Survey⁎⁎	Not indicated
59	Female	45–49	02 Jul	11.0	21 Sep	NR	Contact tracing	No
60	Male	50–54	01 Jul	13.1	29 Sep	NR	Contact tracing	No
61	Male	35–39	21 Aug	13.2	07 Sep	36.8	Healthcare routine testing	No
62	Male	40–44	28 May	13.5	26 Dec	NR	Survey⁎⁎	No
63	Female	50–54	18 Jul	14.5	25 Aug	32.3	Survey⁎⁎	No
64	Female	35–39	04 Jul	14.8	30 Aug	NR	Individual request†	No
65	Female	35–39	18 Jul	15.8	04 Aug	NR	Contact tracing	No
66	Male	45–49	08 Jul	16.0	15 Oct	NR	Healthcare routine testing	No
67	Female	30–34	11 Jul	16.6	29 Jul	36.2	Survey⁎⁎	No
68	Male	60–64	16 Aug	17.3	05 Oct	31.0	Healthcare routine testing	No
69	Male	35–39	25 Aug	17.4	19 Sep	33.7	Survey⁎⁎	Not indicated
70	Male	25–29	02 Aug	17.8	01 Sep	NR	Clinical suspicion§	No††
71	Male	35–39	24 Aug	18.0	11 Oct	NR	Survey⁎⁎	Not indicated
72	Male	35–39	01 Jun	19.7	23 Aug	NR	Survey⁎⁎	No
73	Male	15–19	08 Aug	20.0	12 Sep	34.5	Healthcare routine testing	No
74	Male	50–54	08 Jul	20.1	10 Sep	NR	Clinical suspicion§	No††
75	Male	40–44	09 Jul	20.5	30 Aug	NR	Clinical suspicion§	No††
76	Female	35–39	13 Jul	20.9	27 Aug	NR	Survey⁎⁎	No
77	Male	20–24	22 Aug	20.9	28 Nov	34.9	Survey⁎⁎	No
78	Male	45–49	25 Aug	22.9	25 Sep	34.7	Survey⁎⁎	No
79	Male	50–54	05 Oct	26.9	05 Nov	35.3	Survey⁎⁎	No
80	Male	20–24	10 Aug	28.5	05 Oct	33.0	Survey⁎⁎	No
81	Male	30–34	07 Jul	28.5	21 Aug	34.9	Clinical suspicion§	Yes
82	Male	30–34	26 Aug	30.4	13 Sep	35.3	Survey⁎⁎	Not indicated
83	Male	40–44	28 Jun	31.9	05 Oct	NR	Individual request†	No
84	Male	0–9	01 Jul	32.8	01 Aug	NR	Clinical suspicion§	Yes
85	Male	70–74	21 Jul	33.2	08 Sep	NR	Healthcare routine testing	No
86	Male	40–44	17 Jul	35.8	11 Sep	NR	Survey⁎⁎	No
87	Male	30–34	21 Jul	36.8	12 Sep	NR	Survey⁎⁎	No
88	Male	30–34	01 Jun	37.9	01 Aug	NR	Clinical suspicion§	Yes
89	Female	25–29	06 Jun	38.3	23 Jul	36.0	Survey⁎⁎	No
90	Male	30–34	08 Jul	39.6	23 Jul	34.2	Contact tracing	No
91	Male	30–34	24 Jul	41.9	08 Aug	34.4	Survey⁎⁎	No
92	Female	35–39	09 Nov	43.2	29 Dec	NR	Healthcare routine testing	No
93	Male	25–29	05 Jul	46.0	15 Aug	31.6	Contact tracing	Not indicated
94	Male	20–24	27 Jul	46.2	15 Oct	33.0	Healthcare routine testing	No
95	Male	60–64	28 Sep	47.0	22 Oct	31.3	Survey⁎⁎	No
96	Male	25–29	13 Jul	47.8	28 Jul	NR	Survey⁎⁎	No
97	Male	40–44	13 Jul	48.3	30 Aug	NR	Survey⁎⁎	No
98	Male	35–39	25 Aug	49.4	26 Sep	33.6	Survey⁎⁎	Not indicated
99	Male	25–29	23 Aug	51.7	17 Oct	33.6	Clinical suspicion§	No††
100	Female	10–14	13 Jul	52.4	29 Sep	42.4	Individual request†	Not indicated
101	Male	30–34	13 Jul	54.4	28 Jul	35.9	Survey⁎⁎	No
102	Male	35–39	22 Jul	55.1	21 Oct	37.5	Clinical suspicion§	Yes
103	Male	35–39	05 Jul	56.1	15 Aug	36.2	Survey⁎⁎	No
104	Male	40–44	12 Aug	57.2	21 Oct	36.7	Clinical suspicion§	Yes
105	Male	50–54	27 Aug	57.4	03 Dec	37.3	Healthcare routine testing	No
106	Female	15–19	20 Aug	63.8	24 Oct	NR	Individual request†	No
107	Female	30–34	30 Jul	65.0	29 Sep	36•4	Port of entry‡	No
108	Male	25–29	20 Jul	65.3	22 Aug	NR	Contact tracing	No
109	Male	45–49	22 Jun	66.8	12 Jul	31.3	Contact tracing	No
110	Male	40–44	01 Nov	68.6	26 Dec	NR	Survey⁎⁎	No
111	Female	30–34	18 Jul	73.9	05 Oct	NR	Survey⁎⁎	No
112	Male	60–64	06 Jul	76.5	03 Sep	NR	Healthcare routine testing	No
113	Female	30–34	14 Jul	77.3	15 Aug	37.1	Contact tracing	No
114	Male	45–49	12 Jul	81.5	20 Aug	34.6	Healthcare routine testing	No
115	Male	65–69	18 Aug	85.6	27 Oct	35.7	Port of entry‡	No
116	Male	30–34	26 Jul	92.2	12 Dec	NR	Healthcare routine testing	No
117	Male	40–44	09 Jul	94.1	27 Jul	38.3	Survey⁎⁎	No
118	Male	30–34	01 Sep	97.1	17 Sep	35.8	Healthcare routine testing	No
119	Male	40–44	24 Aug	101.0	28 Nov	33.1	Clinical suspicion§	Yes
120	Male	40–44	21 Jul	101.9	29 Aug	35.0	Survey⁎⁎	No
121	Male	55–59	01 Jul	105.3	17 Aug	NR	Clinical suspicion§	No††
122	Male	35–39	04 Aug	109.2	03 Dec	NR	Survey⁎⁎	No
123	Male	30–34	28 Jul	121.9	20 Aug	35.8	Contact tracing	No
124	Male	35–39	09 Aug	124.4	29 Aug	NR	Individual request†	No
125	Male	40–44	01 Sep	125.3	15 Oct	35.8	Clinical suspicion§	No††
126	Female	60–64	29 Jul	128.0	19 Aug	34.2	Survey⁎⁎	No
127	Male	35–39	11 Aug	141.0	26 Aug	NR	Survey⁎⁎	No
128	Male	35–39	31 Aug	146.0	05 Oct	NR	Clinical suspicion§	Yes
129	Male	30–34	02 Sep	150.0	27 Sep	34.1	Healthcare routine testing	No

Ab, antibody; Asymp, asymptomatic; Ct, cycle threshold; NR, not reported; PCR, polymerase chain reaction.

The table is sorted by antibody test optical density value (antibody titer).

Persons with ID numbers 5, 64, 72, 88, and 127 are reinfection cases that were confirmed by viral genome sequencing.

⁎ Average PCR Ct value over different targets for SARS-CoV-2 genes and/or proteins.

† The category “individual request” refers to testing conducted at a healthcare facility based on the individual's request, often because of some requirement for testing, such as for travel.

‡ The category “port of entry” refers to testing conducted at the border or airport upon return from travel.

§ The category “clinical suspicion” refers to testing conducted at a healthcare facility based on presence of signs or symptoms, or reported history of exposure.

⁎⁎ The category “survey” refers to surveillance random PCR testing campaigns conducted in workplaces and residential areas.

†† The reason for the swab in the hospital record was “clinical suspicion”, but no further details were provided and the person was reported to have no COVID-19 symptoms.

At the time of the reinfection swab, eight cases had records in the severity database. One of these was classified as “severe” and two as “moderate” per WHO classification [23], while the other five were classified as “asymptomatic.” At time of primary infection, 14 cases had records in the severity database, one of whom was classified as “critical”, three as “severe”, five as “moderate”, two as “mild”, and three as “asymptomatic.” For the rest of the reinfection cases, no severity classification was conducted because of minimal or no symptoms to warrant a clinical assessment. For the eight asymptomatic cases above that *had* a severity assessment, the assessment was conducted because of non-COVID-related hospitalization. No deaths were recorded for any of these reinfection cases.

### 3.2 Confirmation of reinfection through viral genome sequencing

**Fig. 2:**
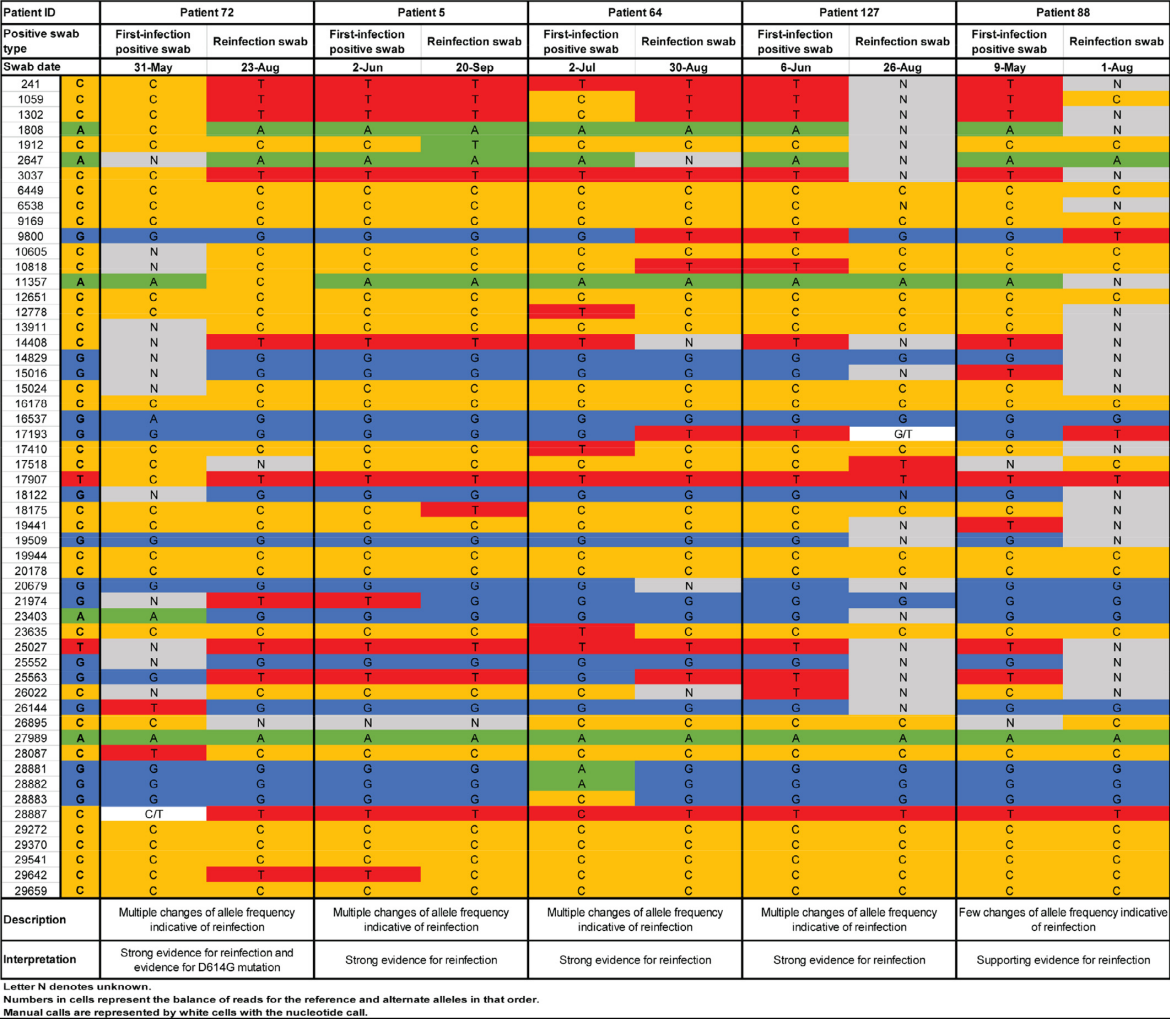
Viral genome sequencing analysis of paired viral specimens of the primary-infection PCR-positive swab and the reinfection PCR-positive swab for five cases with strong or supporting evidence of reinfection. These genomes have been deposited in the public domain [34].

Among the 129 cases with *good* or *some* evidence for reinfection, 62 had records indicating prior diagnosis of a primary infection. Paired specimens of the first-infection PCR-positive swab and the reinfection swab were retrieved in 23 cases. Viral genome sequencing results are summarized in Table 3. Detailed analysis for each genome pair is shown in Fig. 2 and Supplementary Figures S1-S2. Genome sequencing results have been also placed in the public domain [34].

**Table 3:** Results of reinfection confirmation using viral genome sequencing. Viral genome sequencing was conducted only for a subset of cases with *good* or *some* evidence of reinfection, that is, whenever paired samples of the first-infection PCR-positive swab and the reinfection PCR-positive swab were available.

Viral genome sequencing evidence for reinfection	Indication upon comparing each genome pair	N
Insufficient evidence to warrant interpretation	One or two genomes of low quality	7
No evidence for reinfection	One change of allele frequency	1
Shifting balance of quasi-species with no evidence for reinfection	Few changes of allele frequency but not sufficiently indicative of reinfection	6
Strong evidence for no reinfection	Both genomes of high quality yet no significant differences found	4*
Supporting evidence for reinfection	Few changes of allele frequency indicative of reinfection	1
Strong evidence for reinfection	Multiple changes of allele frequency indicative of reinfection	4
**Total**		**23**

PCR, polymerase chain reaction.

⁎ Viral genome sequencing for two patients was performed as part of an earlier study assessing the risk of SARS-CoV-2 reinfection in the cohort of PCR-confirmed infected persons in Qatar [5].

There was insufficient evidence to warrant interpretation for seven sample pairs because of low genome quality. For seven additional pairs, there were one to several changes of allele frequency indicative, at best, of a shifting balance of quasi-species, thus no evidence for reinfection. For four pairs, there was strong evidence for *no reinfection* as both genomes were of high quality, yet no differences were found. Three of these cases had a Ct <30 for the reinfection swab, indicating persistent active infection (Table 2). Two of these cases were reported earlier as part of a case report documenting the existence of prolonged infections [35].

Meanwhile, for one pair, there were few changes of allele frequency offering *supporting evidence for reinfection*. For four other pairs, there were multiple clear changes of allele frequency indicating *strong evidence for reinfection*. One of the latter pairs also documented the presence of the D614G mutation (23,403 bp *A*>*G*) at the reinfection swab—a variant that has progressively replaced the original D614 form [36,37].

In summary, for the 16 cases in which viral genome sequencing evidence was available, five cases were confirmed as reinfections, a confirmation rate of 31.3%. This confirmation rate was similar to that found in our earlier study of reinfection among those with a PCR-confirmed infection at 33.3% [5].

### 3.3 Assessment of risk and incidence rate of reinfection

**Fig. 3:**
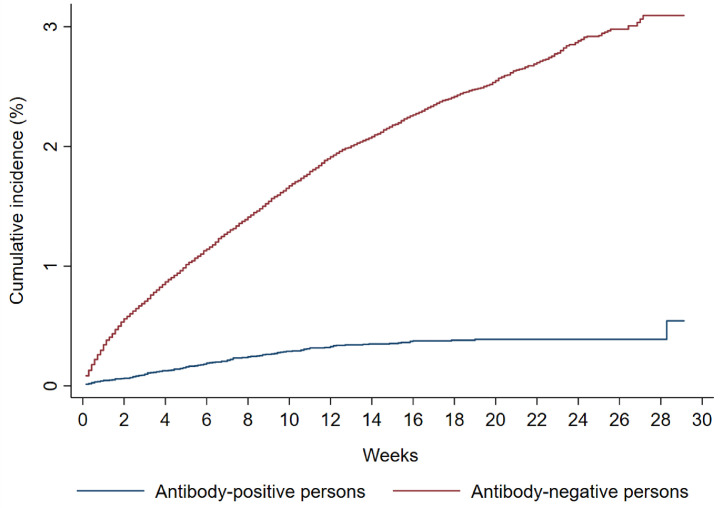
Kaplan-Meier curves showing the cumulative risk (incidence) of documented reinfection and of documented infection with SARS-CoV-2 in the antibody-positive and antibody-negative cohorts, respectively.

The Kaplan–Meier curve for the risk (incidence) of documented reinfection is shown in Fig. 3. Applying the 31.3% confirmation rate obtained through viral genome sequencing, this yielded a cumulative risk of documented reinfection of 0.17% (95% confidence interval (CI): 0.10–0.30%) after 34.6 weeks of follow-up.

The incidence rate of documented reinfection was estimated at 0.66 per 10,000 person-weeks (95% CI: 0.56–0.78). That is 31.3% of 129 reinfection events in a follow-up person-time of 610,832.5 person-weeks.

**Fig. 4:**
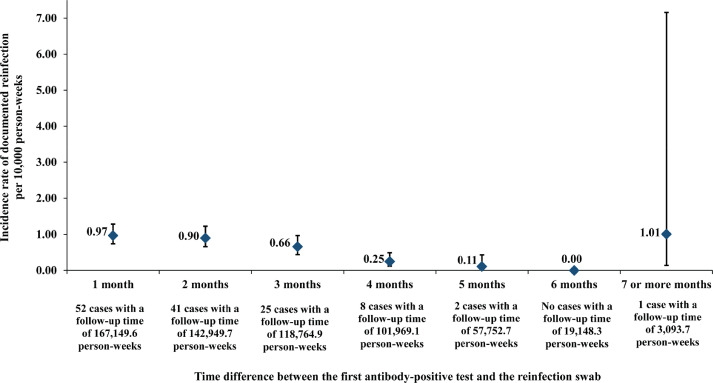
Incidence rate of documented SARS-CoV-2 reinfection versus month of follow-up in the cohort of 43,044 antibody-positive persons. Error bars indicate 95% confidence interval.

Fig. 4 shows the incidence rate of documented reinfection versus month of follow-up in this cohort of antibody-positive persons. There was evidence for a decreasing trend in the incidence rate of reinfection with each additional month of follow-up (Mantel-Haenszel trend analysis p-value: <0.001).

### 3.4 Comparator antibody-negative group and efficacy of natural infection against reinfection

**Fig. 5:**
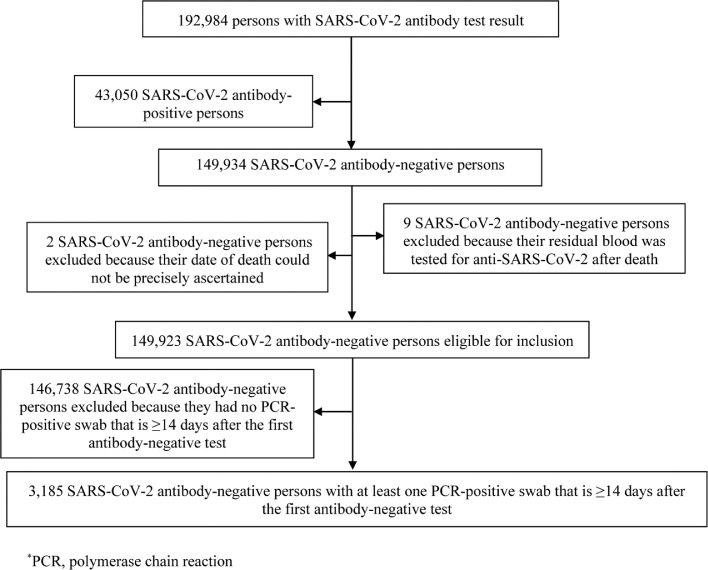
Flowchart describing the process for identifying SARS-CoV-2 incident infections in the complement cohort of antibody-negative individuals.

The complement cohort of all those who tested SARS-CoV-2 antibody-negative included 149,934 individuals. Of those, nine were excluded because their residual blood was tested for SARS-CoV-2 antibodies after death. Two other individuals were excluded because their date of death could not be precisely ascertained. This yielded a retrospective cohort of 149,923 antibody-negative persons to be assessed for SARS-CoV-2 infection incidence (Fig. 5).

This cohort included 75,904 (50.6%) women and 74,019 men (49.4%) of 167 nationalities. Median age was 35 years for women (interquartile range (IQR): 28–47 years) and 39 years for men (IQR: 30–50 years). Individual time of follow-up ranged between 0 days and 45.6 weeks, with a median of 17.0 weeks. These characteristics are similar to those of the antibody-positive cohort apart from the higher proportion of women. The higher proportion of women is a consequence of the fact that men were several-fold more affected than women by the SARS-CoV-2 epidemic and much more likely to be seropositive [12,14,16,17]. The men craft and manual worker population, that comprises 60% of the total population [38], was the most affected segment of the population with a seroprevalence that is much higher than the rest of the population [12,14,16,17].

Of this cohort, 69.2% had received a PCR test with an overall testing frequency of 1.7 tests per person, and of 0.9 tests per person after the first antibody-negative test. Of 149,923 antibody-negative individuals, 3185 individuals had at least one PCR-positive swab ≥14 days after the first antibody-negative test. The Kaplan–Meier curve for the risk (incidence) of documented infection is shown in Fig. 3. The cumulative risk of documented infection was estimated at 3.09% (95% CI: 2.93–3.27%) after 45.6 weeks of follow-up.

The incidence rate of documented infection was estimated at 13.69 per 10,000 person-weeks (95% CI: 13.22–14.14), that is 3185 infections in a follow-up person-time of 2326,572.0 person-weeks.

The *efficacy* of natural infection against reinfection was estimated by comparing the incidence rate of reinfection in the antibody-positive cohort to the incidence rate of infection in the comparator antibody-negative cohort:

$$\text{Efficacy}\;\text{against}\;\text{reinfection}=1-\frac{\text{0.66 per 10,000 person-weeks}}{\text{13.69 per 10,000 person-weeks}},$$

yielding an efficacy estimate of 95.2% (95% CI: 94.1–96.0%).

There were no statistically significant differences in efficacy between women and men, or for those <50 years of age versus those ≥50 years of age. The PCR testing frequency (after the antibody test) among those antibody-positive was lower than that among those antibody-negative, probably reflecting the lower incidence of infection among them. Adjusting the efficacy estimate for differences in testing frequency reduced the estimate to 91.3% (95% CI: 89.4–92.9%), not materially different from the original estimate.

As a sensitivity analysis, the Mantel-Haenszel approach was used to provide another estimate for the efficacy by factoring all PCR testing (after the antibody test) on the combined cohorts stratified by calendar week, that is adjusting for the phase of the epidemic. After applying the viral genome sequencing confirmation rate and excluding PCR-positive cases with weak evidence for reinfection, the efficacy against reinfection was estimated at 92.7% (95% CI: 91.3–93.9%), also not materially different from the original estimate.

## 4 Discussion

The results provide concrete evidence for the presence of reinfection in some individuals with detectable antibodies for SARS-CoV-2 infection, even in some with high antibody titers (Table 2). However, the cumulative risk of documented reinfection was rare, at ~2 per 1000 infected persons, at least for a few months after the first antibody-positive test in the young and international population of Qatar where <9% of the population are ≥50 years of age [11].

There was also no evidence that antibody-positive persons experienced any waning of protective immunity over time, as the incidence rate of reinfection versus month of follow-up did not show an increasing trend over seven months following the first antibody-positive test (Fig. 4). To the contrary, there was a trend of decreasing incidence rate, possibly explained by the (very) slowly declining incidence rate in the wider population of Qatar during this study [15,39], or possibly by strengthening of protective immunity due to repeated exposures that did not lead to established infection. Notably, a recent study from Qatar indicated an association between higher antibody titers and repeated exposures to the virus [17]. Further follow-up of this cohort of antibody-positive persons over time may allow a more long-term assessment of the persistence of protection against reinfection.

Remarkably, the incidence rate of reinfection found here for those with *antibody-confirmed* infection at ~1 per 10,000 person-weeks is very similar to that found for those with *PCR-confirmed* infection, as reported in our earlier reinfection study [5]. This suggests that these two populations are *functionally* similar. Evidence of exposure to SARS-CoV-2, regardless of the biomarker used to assess infection, appears sufficient to indicate protection against reinfection.

These findings are striking, as the epidemic in Qatar has been intense, with half of the population estimated to have acquired this infection at some point since its introduction into Qatar early in 2020 [[14], [15], [16], [17],^39^]. It is highly probable that a proportion of the population has been repeatedly exposed to SARS-CoV-2, but such re-exposures did not lead to more than a limited number of documentable reinfections. Other lines of evidence also support a low frequency of reinfection. The epidemic in Qatar grew rapidly and declined rapidly [15,39], consistent with a susceptible-infected-recovered “SIR” epidemic dynamic in which infection elicits strong immunity against reinfection. No second wave materialized in 2020 following the epidemic peak in May 2020, despite easing of public health restrictions [15,39]. Other studies of reinfection also indicated lower incidence of infection in those antibody-positive or with a prior PCR-confirmed infection [5,[40], [41], [42], [43], [44], [45], [46]], and a study of immunological memory in a cohort of COVID-19 patients indicated durability of the immune response for at least 6–8 months [47].

The study estimated the *efficacy of natural infection against reinfection* at 95.2% by comparing SARS-CoV-2 incidence in those antibody-positive to those antibody-negative. The efficacy can also be estimated by comparing the incidence rate of documented reinfection to the incidence rate of documented infection throughout the epidemic in 2020 that was estimated at ~15 per 10,000 person-weeks [15]. This yielded an efficacy of 95.6%, confirming the above estimate. Remarkably, this efficacy estimate is similar to the efficacy reported for the two mRNA COVID-19 vaccines [18,19], our earlier reinfection study [5], and a study of reinfection in Switzerland [45], but is higher than that reported recently in other studies of reinfection, ranging between 80 and 90% [[40], [41], [42], [43], [44],^46^].

While one reinfection was severe, none were critical or fatal and a large proportion of reinfections were minimally symptomatic (if not asymptomatic) to the extent that they were discovered only incidentally, such as through contact tracing or random testing campaigns/surveys (Table 2). The severity of reinfection was also less than that of primary infection. These findings suggest that reinfections (when they rarely occur) appear well tolerated and no more symptomatic than primary infections.

This study has some limitations. By study design, primary infection was *indirectly* ascertained through serological testing, thereby including only a subset with documented PCR-confirmed primary infections. Having said so, serological testing was based on a high-quality, validated platform, the Roche platform, one of the best available and most extensively used and investigated commercial platforms, with a specificity of at least 99.8% [48,49]. Thus, it is unlikely that misclassified antibody-positives could have biased our findings. We assessed risk of only documented reinfections, but other reinfections may have occurred, but went undocumented, perhaps because of minimal/mild or no symptoms. Recent studies from Denmark and the USA report strong, but still lower protection against reinfection at about 80% [40,46], possibly because of higher diagnosis of asymptomatic and minimally mild reinfections [50]. The follow-up times varied among individuals and a proportion of individuals were followed for a short duration. The antibody-negative cohort had a higher proportion of women than the antibody-positive cohort, due to the differential spread of the infection among women versus men in Qatar [12,14,16,17]. Antigen testing has had limited use in Qatar, and positive results had to be confirmed by PCR testing, therefore it is not likely that reinfections diagnosed through antigen testing could have been missed. Travel history of members of the two cohorts was not available to assess whether there is any loss of follow-up due to travel out of the country.

Viral genome sequencing analysis was possible for only a subset of reinfections, either because primary infection was only identified through antibody testing with no record of earlier PCR testing, or because the reinfection swab could not be retrieved. Reinfections were confirmed by noting differences in the viral genome between the primary infection and the reinfection. While not likely, it is theoretically possible that these differences may have occurred due to within-host evolution of the virus, as in the context of a prolonged infection [35,51].

Unlike in blinded, randomized clinical trials, the two observational cohorts of those antibody-positive and antibody-negative were not randomized. However, most of those antibody-positive or antibody-negative were not aware of their antibody status; thus, it is not likely that awareness of antibody status could have biased the results. In a small proportion of those antibody-negative, there was a record of a prior PCR-positive result. Nonetheless, excluding those with a prior PCR-positive result did not affect the estimated protection against reinfection, which was estimated once more at 95.0% (95% CI: 93.9–95.9). During the course of follow-up, some of those antibody-negative may have developed antibodies, but the infection was not documented by PCR, and so misclassified as controls. It is also possible that those antibody-positive may have been at higher risk of reinfection because of a higher number of contacts, or may have been at lower risk of reinfection because their peers within their social network were also previously infected and thereby less likely to be infected and to transmit the infection to them. The overall potential effect of these departures from the hypothetical ideal of a randomized clinical trial is perhaps an overestimation, rather than underestimation of the incidence of reinfection, thereby affirming the conclusion of the rarity of reinfections.

In conclusion, SARS-CoV-2 reinfection was investigated in a large cohort of antibody-positive individuals who were followed for as long as 35 weeks. While the study documented some reinfections, they constitute a rare phenomenon, with natural infection eliciting protection against reinfection with an efficacy of ~95%. This points to development of robust immunity following primary infection, which lasts for at least seven months. These findings suggest that prioritizing vaccination for those who are antibody-negative, as long as doses of the vaccine remain in short supply, could enhance the health, societal, and economic gains attained by vaccination.

## Funding

The authors are grateful for support from the Biomedical Research Program, the Biostatistics, Epidemiology, and Biomathematics Research Core, and the Genomics Core, all at Weill Cornell Medicine-Qatar, as well as for support provided by the Ministry of Public Health and Hamad Medical Corporation. The authors are also grateful for support from the Qatar Genome Programme for supporting the viral genome sequencing. The statements made herein are solely the responsibility of the authors.

## Author contributions

LJA conceived and designed the study, led the statistical analyses, and co-wrote the first draft of the article. HC contributed to study design, performed the data analyses, and co-wrote the first draft of the article. HC and LJA had access to raw data. JAM led the viral genome sequencing analyses and AAA, YAM, and SY conducted these analyses. All authors contributed to data collection and acquisition, database development, discussion and interpretation of the results, and to the writing of the manuscript. All authors have read and approved the final manuscript.

## Data sharing

All relevant data are available within the manuscript and its supplementary materials.

## Declaration of Competing Interest

We declare no competing interests.

## Appendix Supplementary materials

Image, application 1
