# Supplementary material for: SARS-CoV-2 antibody-positivity protects against reinfection for at least seven months with 95% efficacy
Source: eClinicalMedicine. 2021 Apr 28;35:100861. doi: 10.1016/j.eclinm.2021.100861 (PMC8079668; doi:10.1016/j.eclinm.2021.100861)
Supplement: Supplementary file 1 [file mmc1.docx]

**Supplementary Appendix**

**SARS-CoV-2 antibody-positivity protects against reinfection for at least seven months with 95% efficacy**

Laith J. Abu-Raddad, PhD, Hiam Chemaitelly, MSc, Peter Coyle, MD, Joel A. Malek, PhD, Ayeda A. Ahmed, BSc, Yasmin A. Mohamoud, MSc, Shameem Younuskunju, MSc, Houssein H. Ayoub, PhD, Zaina Al Kanaani, PhD, Einas Al Kuwari, MD, Adeel A. Butt, MBBS MS, Andrew Jeremijenko, MD, Anvar Hassan Kaleeckal, MSc, Ali Nizar Latif, MD, Riyazuddin Mohammad Shaik, MSc, Hanan F. Abdul Rahim, PhD, Gheyath K. Nasrallah, PhD, Hadi M. Yassine, PhD, Mohamed G. Al Kuwari, MD, Hamad Eid Al Romaihi, MD, Mohamed H. Al-Thani, MD, Abdullatif Al Khal, MD, and Roberto Bertollini, MD

^*^Address reprints requests or correspondence to Professor Laith J. Abu-Raddad, Infectious Disease Epidemiology Group, World Health Organization Collaborating Centre for Disease Epidemiology Analytics on HIV/AIDS, Sexually Transmitted Infections, and Viral Hepatitis, Weill Cornell Medicine - Qatar, Qatar Foundation - Education City, P.O. Box 24144, Doha, Qatar. Telephone: +(974) 4492-8321. Fax: +(974) 4492-8333. E-mail: [lja2002@qatar-med.cornell.edu](mailto:lja2002@qatar-med.cornell.edu)

**Table of Contents**

**Table S1.** STROBE statement checklist for cohort studies**3**

**Text S1.** Details of the viral genome sequencing methods**4**

**Figure S1.** Viral genome sequencing analysis of the paired viral specimens of the primary-infection PCR-positive swab and the reinfection PCR-positive swab for the eleven cases with evidence not supporting occurrence of reinfection**6**

**Figure S2.** Viral genome sequencing analysis of the paired viral specimens of the primary-infection PCR-positive swab and the reinfection PCR-positive swab for the seven cases with insufficient genetic evidence to confirm the reinfection**9**

**References 10**

***Table S1*. STROBE statement checklist for cohort studies.**

|  | Item No | Recommendation | Page No |
| --- | --- | --- | --- |
| **Title and abstract** | 1 | (*a*) Indicate the study’s design with a commonly used term in the title or the abstract | 1-2 |
|  |  | (*b*) Provide in the abstract an informative and balanced summary of what was done and what was found | 2-3 |
| Introduction | | | |
| Background/rationale | 2 | Explain the scientific background and rationale for the investigation being reported | 6 |
| Objectives | 3 | State specific objectives, including any prespecified hypotheses | 6-7 |
| Methods | | | |
| Study design | 4 | Present key elements of study design early in the paper | 7 |
| Setting | 5 | Describe the setting, locations, and relevant dates, including periods of recruitment, exposure, follow-up, and data collection | 7, 9, 11 |
| Participants | 6 | (*a*) Give the eligibility criteria, and the sources and methods of selection of participants. Describe methods of follow-up | 7, 9, 11 |
|  |  | (*b*) For matched studies, give matching criteria and number of exposed and unexposed | NA |
| Variables | 7 | Clearly define all outcomes, exposures, predictors, potential confounders, and effect modifiers. Give diagnostic criteria, if applicable | 10-11 |
| Data sources/ measurement | 8* | For each variable of interest, give sources of data and details of methods of assessment (measurement). Describe comparability of assessment methods if there is more than one group | 7-11 |
| Bias | 9 | Describe any efforts to address potential sources of bias | 9-10 |
| Study size | 10 | Explain how the study size was arrived at | 7-8 |
| Quantitative variables | 11 | Explain how quantitative variables were handled in the analyses. If applicable, describe which groupings were chosen and why | 10-11 |
| Statistical methods | 12 | (*a*) Describe all statistical methods, including those used to control for confounding | 9-11 |
|  |  | (*b*) Describe any methods used to examine subgroups and interactions | NA |
|  |  | (*c*) Explain how missing data were addressed | NA |
|  |  | (*d*) If applicable, explain how loss to follow-up was addressed | 10-11 |
|  |  | (*e*) Describe any sensitivity analyses | 10-11 |
| Results | | |  |
| Participants | 13* | (a) Report numbers of individuals at each stage of study—eg numbers potentially eligible, examined for eligibility, confirmed eligible, included in the study, completing follow-up, and analysed | 12, 15, Figs 1 & 4 |
|  |  | (b) Give reasons for non-participation at each stage | 12, 15, Figs 1 & 5 |
|  |  | (c) Consider use of a flow diagram | Figs 1 & 5 |
| Descriptive data | 14* | (a) Give characteristics of study participants (eg demographic, clinical, social) and information on exposures and potential confounders | 12, 15 |
|  |  | (b) Indicate number of participants with missing data for each variable of interest | 12, 15 |
|  |  | (c) Summarise follow-up time (eg, average and total amount) | 12, 15 |
| Outcome data | 15* | Report numbers of outcome events or summary measures over time | 12-16 and Fig 4 |
| Main results | 16 | (*a*) Give unadjusted estimates and, if applicable, confounder-adjusted estimates and their precision (eg, 95% confidence interval). Make clear which confounders were adjusted for and why they were included | 12-16 |
|  |  | (*b*) Report category boundaries when continuous variables were categorized | NA |
|  |  | (*c*) If relevant, consider translating estimates of relative risk into absolute risk for a meaningful time period | 14-16, Fig 3 |
| Other analyses | 17 | Report other analyses done—eg analyses of subgroups and interactions, and sensitivity analyses | 17 |
| Discussion | | | |
| Key results | 18 | Summarise key results with reference to study objectives | 17 |
| Limitations | 19 | Discuss limitations of the study, taking into account sources of potential bias or imprecision. Discuss both direction and magnitude of any potential bias | 19-20 |
| Interpretation | 20 | Give a cautious overall interpretation of results considering objectives, limitations, multiplicity of analyses, results from similar studies, and other relevant evidence | 17-20 |
| Generalisability | 21 | Discuss the generalisability (external validity) of the study results | 19-20 |
| Other information | | | |
| Funding | 22 | Give the source of funding and the role of the funders for the present study and, if applicable, for the original study on which the present article is based | 11, 22 |

Figs, figures; NA, not applicable.

***Text S1*. Details of the viral genome sequencing methods.**

Viral RNA was extracted using Quick-RNA Viral Kit (Zymo Research, Irvine, USA; Cat. No. R1041) and eluted in 30ul of nuclease-free water. RNA quality was assessed with real-time quantitative polymerase chain reaction (RT-qPCR) using SARS-CoV-2 (2019-nCoV) CDC qPCR Probe Assay Research Use Only (RUO) kit (Integrated DNA Technologies, USA; Cat number 10006713) and Luna Universal Probe One-Step RT-qPCR Kit (New England BioLabs, USA; Cat number E3006E) on Applied Biosystems 7500 Fast Real-Time PCR instrument (Applied Biosystems, CA, USA).

Next-generation sequencing (NGS) library construction was performed using CleanPlex SARS-CoV-2 Panel (Paragon Genomics, USA; SKU: 918012). Gel-size selection on a 3% agarose gel was utilized to prevent formation of adapter dimers. NGS libraries were quantified using KAPA Library Quantification Kit (Roche, USA; KK4824), and normalized, pooled, and sequenced on an Illumina MiSeq instrument using a paired-end 150bp kit (Illumina, USA; MS-102-2002). All procedures were implemented following manufacturers’ protocols.

Raw sequences were processed with CUTADAPT (v2·10)^1^ to exclude the contaminating adapter sequences. Adapter trimming was performed using parameters -g CCTACACGACGCTCTTCCGATCT **-a** AGATCGGAAGAGCACACGTCTGAA **-A** AGATCGGAAGAGCGTCGTGTAGG **-G** TTCAGACGTGTGCTCTTCCGATCT **-e** 0·1 **-O** 9 **-m** 50 **-n** 2. Only paired reads with minimum length of 50bp were retained for analysis. The latter filtered reads were aligned to SARS-CoV-2 reference genome (NC_045512) using BWA-MEM.^2^ FGBIO (v1·3·0) was subsequently used to remove PCR primer sequences from the resulting BAM file.

Variant calling and genotyping were performed with VarScan multi-sample mpileup^3^ with the pileup file generated using SAMTOOLS mpileup (v1·10)^4^ with --min-BQ 20 and --min-MQ 20 parameters. The mpileup2snp function of VarScan was then applied with the filtering parameters --min-var-freq 0·2, --min-coverage 5, and --min-avg-qual 20, to generate the final VCF file.

***Figure S1*. Viral genome sequencing analysis of the paired viral specimens of the primary-infection PCR-positive swab and the reinfection PCR-positive swab for the eleven cases with evidence not supporting occurrence of reinfection. These genomes have been deposited in the public domain.^5^**

1. **Two individuals with *strong evidence for no reinfection*** (using viral genome sequencing conducted in this study)


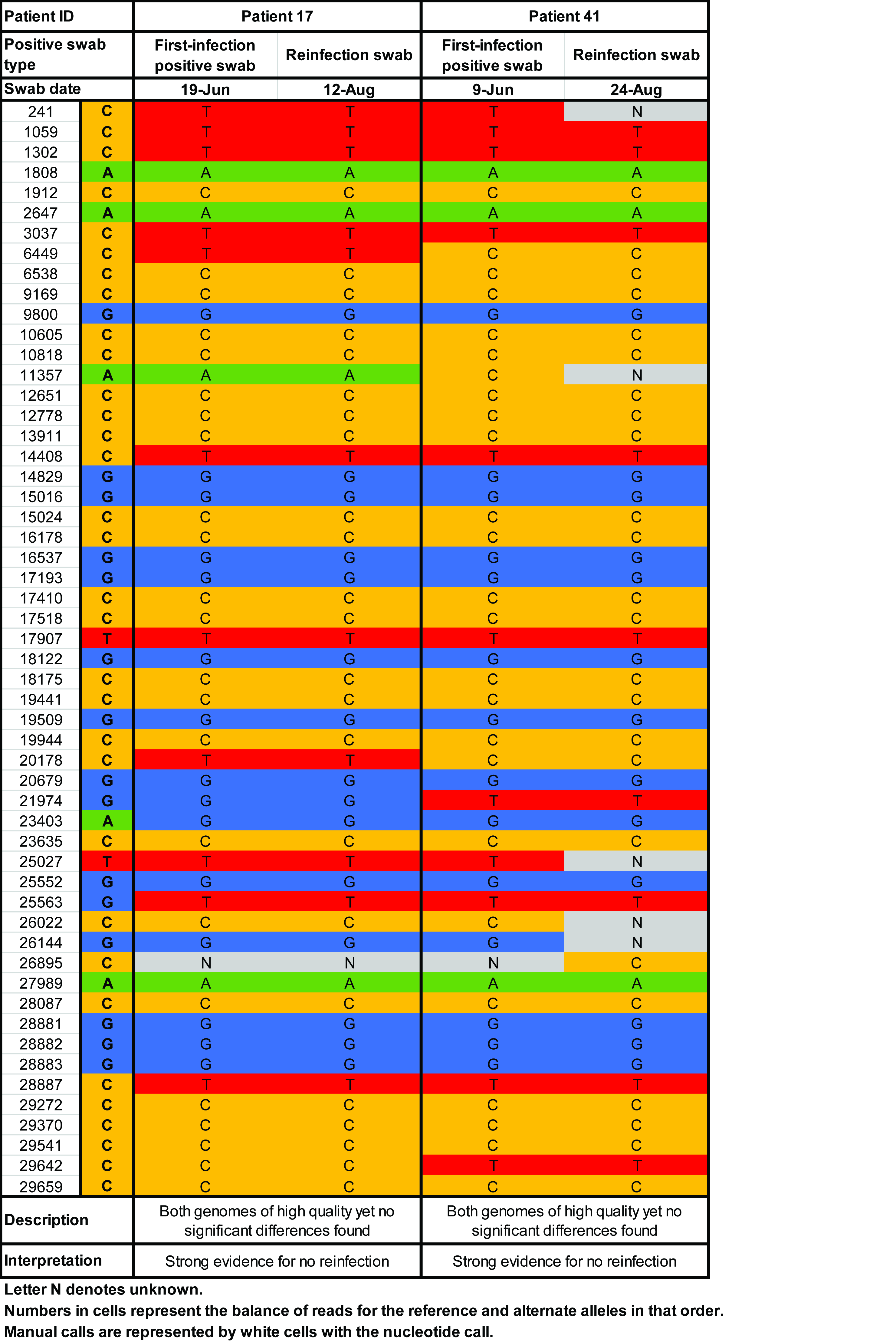


1. **Two individuals with *strong evidence for no reinfection*** (using viral genome sequencing conducted in an earlier study^6^)

**
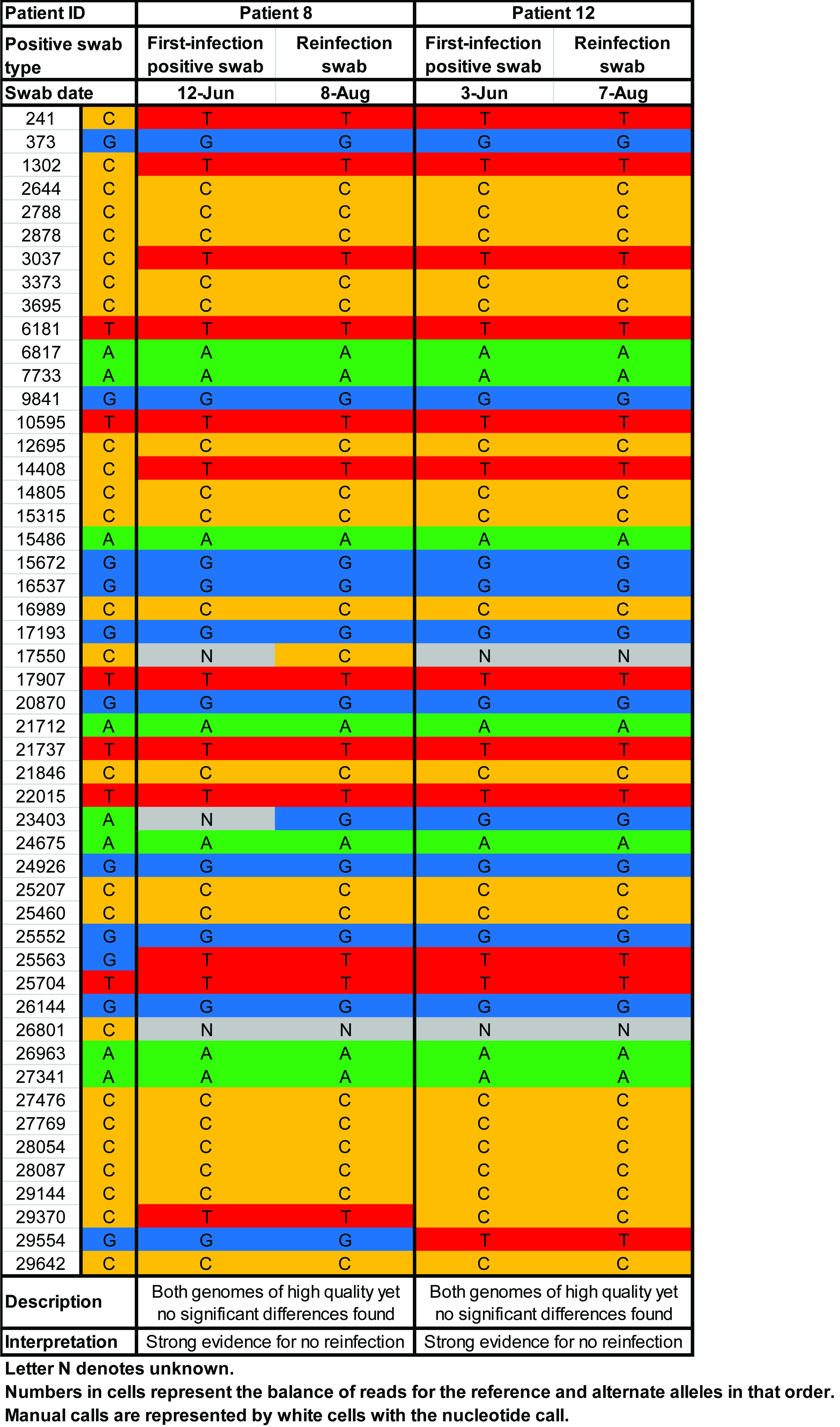
**

1. **Seven individuals with *no evidence for reinfection***

**
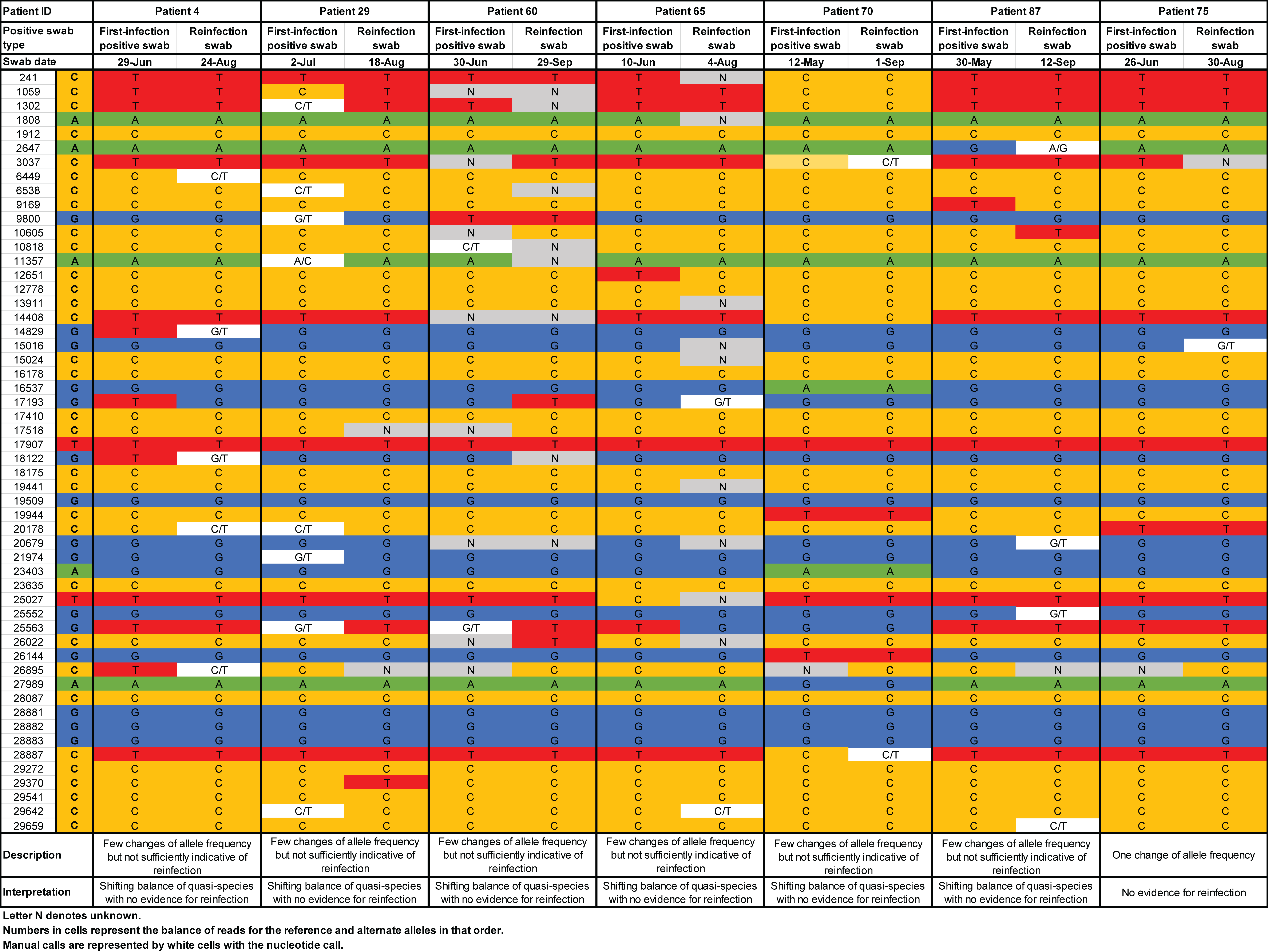
**

***Figure S2*. Viral genome sequencing analysis of the paired viral specimens of the primary-infection PCR-positive swab and the reinfection PCR-positive swab for the seven cases with insufficient genetic evidence to confirm the reinfection. These genomes have been deposited in the public domain.^5^**


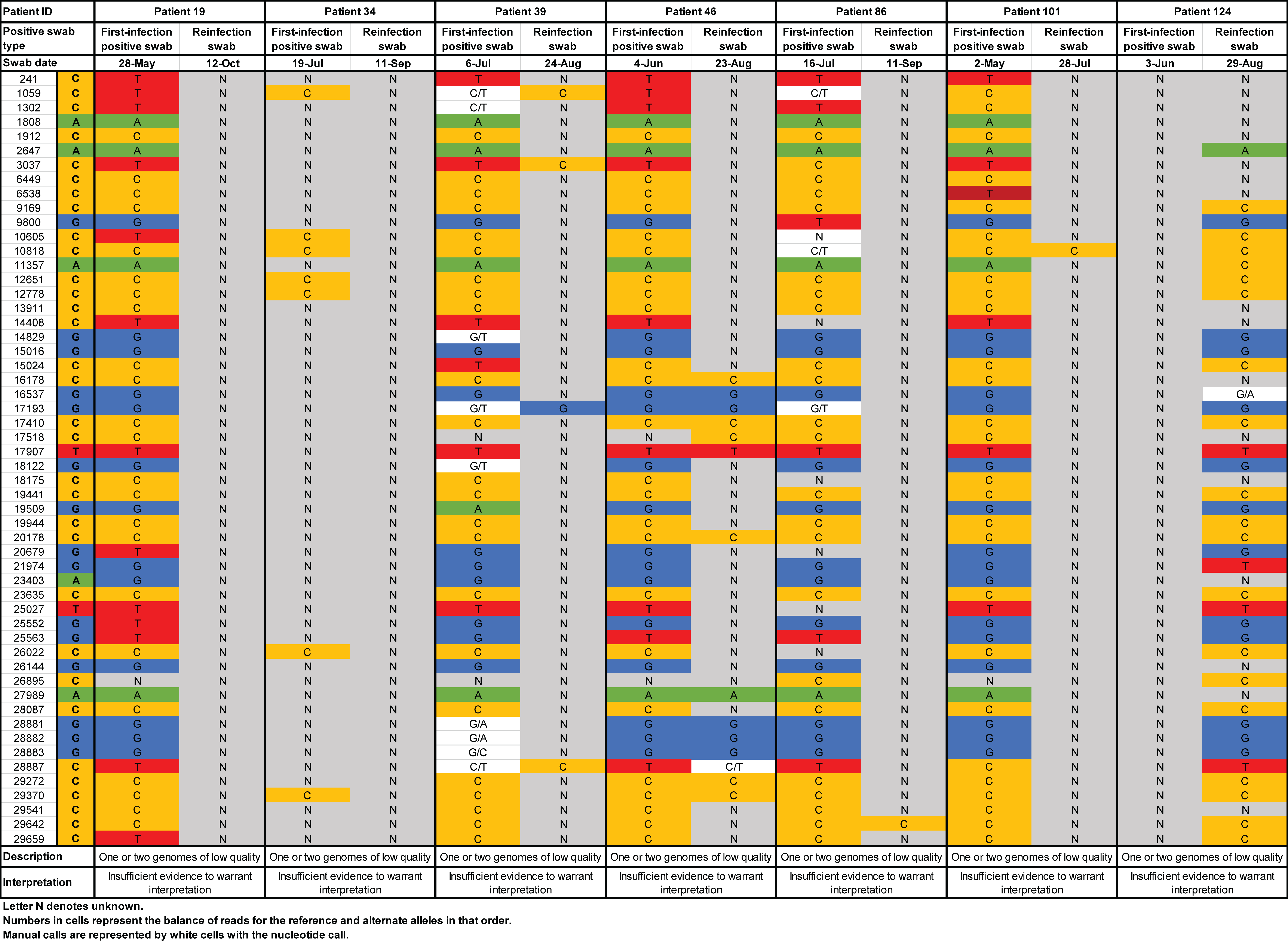


**References**

1. Martin M. Cutadapt removes adapter sequences from high-throughput sequencing reads. ISSN 2226-6089. Available at: <<http://journal.embnet.org/index.php/embnetjournal/article/view/200>>. Date accessed: 17 sep. 2020. doi:<https://doi.org/10.14806/ej.17.1.200>. *EMBnetjournal* 2011; **17**(1): 10-2.

2. Li H, Durbin R. Fast and accurate short read alignment with Burrows-Wheeler transform. *Bioinformatics* 2009; **25**(14): 1754-60.

3. Koboldt DC, Chen K, Wylie T, et al. VarScan: variant detection in massively parallel sequencing of individual and pooled samples. *Bioinformatics* 2009; **25**(17): 2283-5.

4. Li H, Handsaker B, Wysoker A, et al. The Sequence Alignment/Map format and SAMtools. *Bioinformatics* 2009; **25**(16): 2078-9.

5. Weill Cornell Medicine in Qatar. SARS-CoV-2 reinfection in a cohort of 43,000 antibody-positive individuals followed for up to 35 weeks. <https://www.ncbi.nlm.nih.gov/bioproject/PRJNA698478/>. <https://www.ncbi.nlm.nih.gov/bioproject/?term=PRJNA699251/>. 2021.

6. Abu-Raddad LJ, Chemaitelly H, Malek JA, et al. Assessment of the risk of SARS-CoV-2 reinfection in an intense re-exposure setting. *Clinical Infectious Diseases* 2020. ciaa1846. doi: 10.1093/cid/ciaa1846.
